# Cancer Burden in Neighborhoods With Greater Racial Diversity and Environmental Burden

**DOI:** 10.1001/jamanetworkopen.2025.16740

**Published:** 2025-06-20

**Authors:** Jessica R. Bobbitt, Fangzhou Liu, Ruth A. Keri, Jennifer Cullen

**Affiliations:** 1 Department of Pathology, School of Medicine, Case Western Reserve University, Cleveland, Ohio; 2 Department of Population and Quantitative Health Sciences, School of Medicine, Case Western Reserve University, Cleveland, Ohio; 3 Case Comprehensive Cancer Center, Cleveland, Ohio; 4 Department of Cancer Biology, Cleveland Clinic Lerner Research Institute, Cleveland, Ohio; 5 Houston Methodist Dr Mary and Ron Neal Cancer Center, Houston, Texas

## Abstract

**Importance:**

Ohio has among the nation’s highest environmental burden and disproportionately higher lung or bronchus cancer incidence rates.

**Objective:**

To examine (1) the association between environmental burden and cancer, (2) the association between minoritized population and cancer, and (3) the association of environmental burden-minoritized population jointly with cancer.

**Design, Setting, and Participants:**

An area-level cohort study examining mean annual age-adjusted cancer incidence rates in Ohio from 2011 to 2020. Data were analyzed between May and September 2024.

**Exposures:**

Environmental burden and racial and ethnic composition of Ohio residents from the Centers for Disease Control and Prevention’s Environmental Justice Index.

**Main Outcomes and Measures:**

Age-adjusted cancer incidence rates (overall- and lung or bronchus–specific) were examined using the Ohio Cancer Incidence Surveillance System.

**Results:**

Of 2952 total census tracts in the state of Ohio, 2719 (92.1%) contained patients with any cancer and 2510 (85.0%) contained patients with lung or bronchus cancer. In the census tracts with lung or bronchus cancer, patients had a median (IQR) age of 69 (61-76) vs 65 (56-74) years overall, 1328 (52.9%) were male vs 1360 (50.1%) overall, and the median (IQR) percentage of the population that was minoritized was 11.3% (5.40%-29.0%) vs 12.0% (5.60%-32.1%) overall. For any cancer, individuals in the lowest quartile of environmental burden experienced 414.2 cases per 100 000, whereas individuals in the highest quartile experienced 479.5 cases, a difference of 61.1 (95% CI, 47.89-75.42) cases per 100 000 (P < .001). Similarly, in areas with the highest proportion of racially and ethnically minoritized individuals (Q4 vs Q1), overall cancer (81.27 per 100 000; 95% CI, 70.08-92.45; *P* < .001) and lung or bronchus cancer (24.99 per 100 000; 95% CI, 21.57-28.40; *P* < .001) significantly increased. This corresponded to a 20% (483.8 vs 402.5 cases) and 43% (82.6 vs 57.8 cases) increase in overall and lung or bronchus cancer incidence rates, respectively. Census tracts experiencing both high environmental burden and racial and ethnic diversity (Q4:Q4) had significantly higher cancer rates compared with all other regions, both overall (mean [SD], 508.5 [146.2] per 100 000; 95% CI, 491.3-525.7; vs 425.4 [101.4] per 100 000; 95% CI, 421.4-429.5; *P* < .001) and a 58.9% higher incidence of lung and bronchus cancer (mean [SD], 92.4 [41.6] per 100 000; 95% CI, 87.3-97.5; vs 58.2 [20.9] per 100 000; 95% CI, 55.3-61.0; *P* < .001).

**Conclusions and Relevance:**

This cohort study found that cancer incidence rates were associated with environmental burden and with racial and ethnic composition, suggesting the need for sustained community interventions in minoritized census regions with high environmental burden.

## Key Points

**Question:**

Is there an association between environmental burden, racial residential composition, and rates of overall or lung or bronchus cancer incidence rates in Ohio neighborhoods?

**Findings:**

In this area-level cohort study of 2719 Ohio census tracts, the greatest overall cancer incidence was observed in census tracts with both the highest environmental burden coupled with the highest proportion of minoritized residents. These study associations were statistically significant when examining overall cancer incidence as well as lung or bronchus cancer incidence.

**Meaning:**

Environmental burden may disproportionately be associated with cancer incidence rates in minoritized communities, and these regions should be prioritized for environmental remediation efforts.

## Teaser

This cohort study evaluates the association between environmental burden and racial residential composition in Ohio census tracts and combined association with overall and lung or bronchus-specific cancer incidence rates.

## Introduction

Lung or bronchus cancer is the most commonly diagnosed cancer and the leading cause of cancer-related deaths worldwide.^1,2^ However, the burden of lung or bronchus cancer is not equally shared, and highly vulnerable populations are more likely to be diagnosed with and die from this disease.^3,4^ These disparities are even more pronounced in the state of Ohio, which has a disproportionately high rate of cancer incidence and mortality, particularly for lung or bronchus cancer, compared with the nation.^5,6^ The work described herein seeks to understand potential factors associated with these disparities.

Lung or bronchus cancer is largely associated with modifiable risk factors. While the impact of tobacco smoke is well defined,^7,8,9,10,11,12^ the role of other risk factors, such as environmental contamination, is relatively understudied.^12,13^ However, the US Centers for Disease Control and Prevention (CDC) recently compiled several measures of environmental exposure and indicators of environmental burden.^14^ An initial study revealed regions of the country with disproportionately high environmental burden, including a portion of the Midwest encompassing most of Ohio.^14^ They then assembled these data along with social and health outcomes to create the Environmental Justice Index (EJI) database.^15^ An important strength of this dataset is the exploration of environmental, social, and health outcomes at the census tract level, which represents a more homogenous geographic region than the county level, which is a commonly modeled geographic unit of observation. The creation of this dataset has already begun to facilitate the understanding of a range of environmental exposures on public health,^16,17,18,19^ but has not yet been examined for association with overall cancer, or more specifically, with lung or bronchus cancer incidence. Moreover, it is known that non-Hispanic Black and Hispanic individuals tend to experience greater cancer mortality,^20^ underscoring the importance of understanding the reasons for these disparities.

We hypothesized that the high environmental burden in Ohio would be associated with higher cancer burden and lung or bronchus cancer disparities. The study described herein examines the association between area-level environmental exposures, area-level racial and ethnic composition, and cancer incidence in the state of Ohio. The primary study aim was to examine the independent and joint associations between census tract–level environmental burden and racial and ethnic composition on census tract–level, mean annual age-adjusted, overall cancer incidence rates in Ohio; a secondary study aim was to examine these associations for mean annual age-adjusted lung or bronchus cancer incidence rates.^12,13,21^

## Methods

### Independent Variables

Demographic information at the census tract level, including sex (male or female) and age (in years), was extracted from the Ohio Cancer Incidence Surveillance System (OCISS) database. Data on racial and ethnic composition at the census-tract level were collected from the EJI database,^15^ which reports 2015 to 2019 Population-Level Analysis and Community Estimates of health data from the CDC. The percentage of the population that was minoritized was also abstracted from the EJI database, defining minoritized as the percentage of all persons except White, non-Hispanic individuals, in a census tract. Use of OCISS data was reviewed and approved by the Ohio Department of Health institutional review board, and patients provided written informed consent to OCISS. Because data were deidentified, this study did not require review, in accordance with 45 CFR §46. This study adhered to the Strengthening the Reporting of Observational Studies in Epidemiology (STROBE) reporting guidelines.

Environmental data were collected from the EJI database from the CDC.^15^ A subset of EJI measures were of interest, including the EJI variable subset referred to as theme 2, which includes the following 6 key factors: proximity to a superfund site, a toxic release inventory site, storage and disposal sites, risk management plan sites, coal mines, and lead mines. The CDC ranked each census tract based on these 6 factors and calculated an overall score by summing the ranked scores. We then used those census-tract level environmental burden scores in our analysis.

### Outcome of Interest

All Ohio census tracts were assessed for mean annual age-adjusted cancer incidence rates, obtained from the OCISS database^22^ for the period 2011 to 2020. Following guidelines from the Ohio Department of Health, census tracts with less than 11 total cancer cases had to be excluded from the analysis to prevent patient identification. This resulted in 2719 of 2952 census tracts (92%) included for analysis of overall cancer incidence, and 2510 census tracts (85%) were retained for analysis of lung or bronchus cancer.

### Statistical Analysis

The OCISS database contains individual-level data on cancer incidence.^22^ We first used geographical codes to group these individual cancer records into the census tract level. We subsequently calculated the age-adjusted incidence rates standardized to the 2000 US census population. This method of age adjustment allows for the regional comparison of cancer incidence rates, while accounting for variations in age distributions. Age-adjusted incidence rates for overall cancer combined and lung or bronchus cancer were evaluated separately. Rates were then averaged across the study time period (2011-2020).

ArcGIS Pro version 3.3.2 was then used to geographically map census-tract level scores for overall cancer incidence, lung or bronchus cancer incidence, environmental burden, and minoritized status in Ohio, using a quartile (Q) classification method. Geographical identifiers were used to concatenate each variable with spatial matching of Ohio census tracts. These maps represent the 2510 census tracts with data for all 4 variables, with suppression regions displayed in white.

A sizable number of census tracts (389 tracts [14%]) had environmental burden values equal to 0, requiring data categorization due to significant data skew and nonnormality. For the environmental burden variable, all non-0 observations were divided into quartiles and a fifth category that combined all census tracts with environmental burden values equal to 0. For minoritized status, there were 8 census tracts (0.29%) with values observed as 0. Therefore, the minoritized status variable was divided into quartiles, with 0 values included in lowest quartile. This approach resulted in quasiordinal variables to reduce the impact of skewness due to data peaks at values of 0. Linear regression models were then used to examine the association between age-adjusted cancer incidence rates (ie, overall or lung or bronchus-specific) as a function of environmental burden and minoritized percentage. For each model, the slope coefficients (β) were estimated, and 2-sided statistical tests were conducted to determine whether the coefficients were significantly different from each other, with statistical significance set at *P* ≤ .05.

Census tracts were grouped based on their quartile rankings for both minoritized status and environmental burden, resulting in 16 unique combinations (eg, Q1:Q1, Q1:Q2, ..., Q4:Q4). A 2-way analysis of variance was conducted to assess overall differences in cancer incidence rates across these quartile combinations. In a separate analysis, a Welch *t *test was used to compare tracts in the highest quartile for both variables (Q4:Q4) against all other quartile combinations. Statistical significance for both tests was defined as P ≤ .05.

Diagnostic residual and Q-Q plots were performed to ensure that the assumptions of linear regression (linearity, homoscedasticity, and normality) were met. No significant deviations from these assumptions were detected. Cook distance was used to evaluate the association between individual data points, and no significant outliers were identified. We used R version 4.4.0 (R Project for Statistical Computing) version 2024.04.2 for all analyses. Data were analyzed between May and September 2024.

## Results

### Descriptive Characteristics

**Figure 1.**
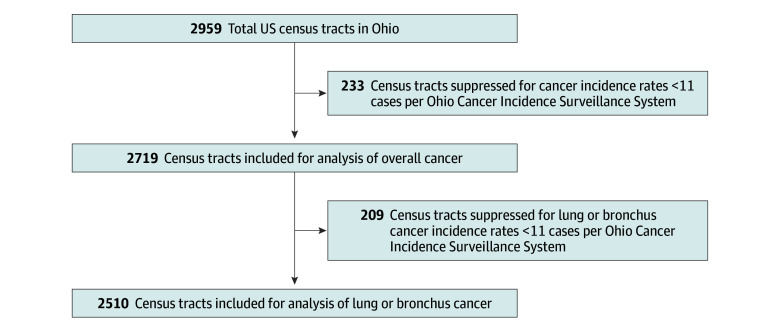
Census Tract Selection

Of 2952 total Ohio census tracts, 2719 were analyzed for their rates of overall cancer, while 2510 were analyzed for their rates of lung or bronchus cancer, representing 92.1% and 85.0% of total tracts, respectively (Figure 1). Within the 2719 and 2510 census tracts, the median (IQR) population age was 65 (56-74) and 69 (61-76) years, respectively; 1360 (50.1%) and 1328 (52.9%) were male, respectively; and the median (IQR) percentage of the population that was minoritized was 12% (5.60%-32.1%) and 11.3% (5.40-29.0), respectively. From 2011 to 2020, the median (IQR) age-adjusted overall cancer incidence rate in those 2719 census tracts was 411.1 (371.8-470.0) cases per 100 000 people per year (Table). The median (IQR) age-adjusted lung or bronchus cancer incidence rate for the 2510 census tracts was 57.8 (43.7-78.8) cases per 100 000 people per year (Table).

**Table.** Descriptive Statistics for Census Tracts in Ohio From 2011 to 2020

Characteristic	Participants	
	Overall cancer (n = 2719)	Lung/bronchus cancer (n = 2510)
Age-adjusted cancer incidence, median (IQR) [range]^a^	411.1 (371.8-470.0) [191.4-2661]	57.80 (43.73-78.80) [16.30-391.7]
Environmental burden, median (IQR) [range]	0.86 (0.49-1.64) [0.00-4.81]	0.86 (0.49-1.11) [0.00-4.81]
Minoritized percentage, median (IQR) [range]	12.0 (5.60-32.1) [0.00-100]	11.3 (5.40-29.0) [0.00-100]
Sex, No. (%)		
Male	1360 (50.1)	1328 (52.9)
Female	1359 (49.9)	1182 (47.1)
Age, median (IQR) [range], y	65 (56-74) [0-100]	69 (61-76) [2-103]

^a^ Cancer incidence represents the mean annual rate per 100 000, age-adjusted to the 2000 US standard population.

The range of the environmental burden variable^23^ for Ohio spans the national range (0.00-4.81), as census tracts in Ohio hold the highest scores across the country, and the median (IQR) score in Ohio is 0.86 (0.49-1.64) (Table). In Ohio, the percentage of a census tract composed of racially or ethnically minoritized individuals^15^ is highly variable, with a range of 0%-100%. For the census tracts included in the analysis of overall cancer, the median (IQR) rate of minoritized individuals was 12.0% (5.60%-32.1%), and for the census tracts analyzed for lung or bronchus cancer, the median (IQR) rate of minoritized individuals was 11.3% (5.40%-29.0%) (Table).

### Environmental Burden and Minoritized Status Association With Cancer Incidence

**Figure 2.**
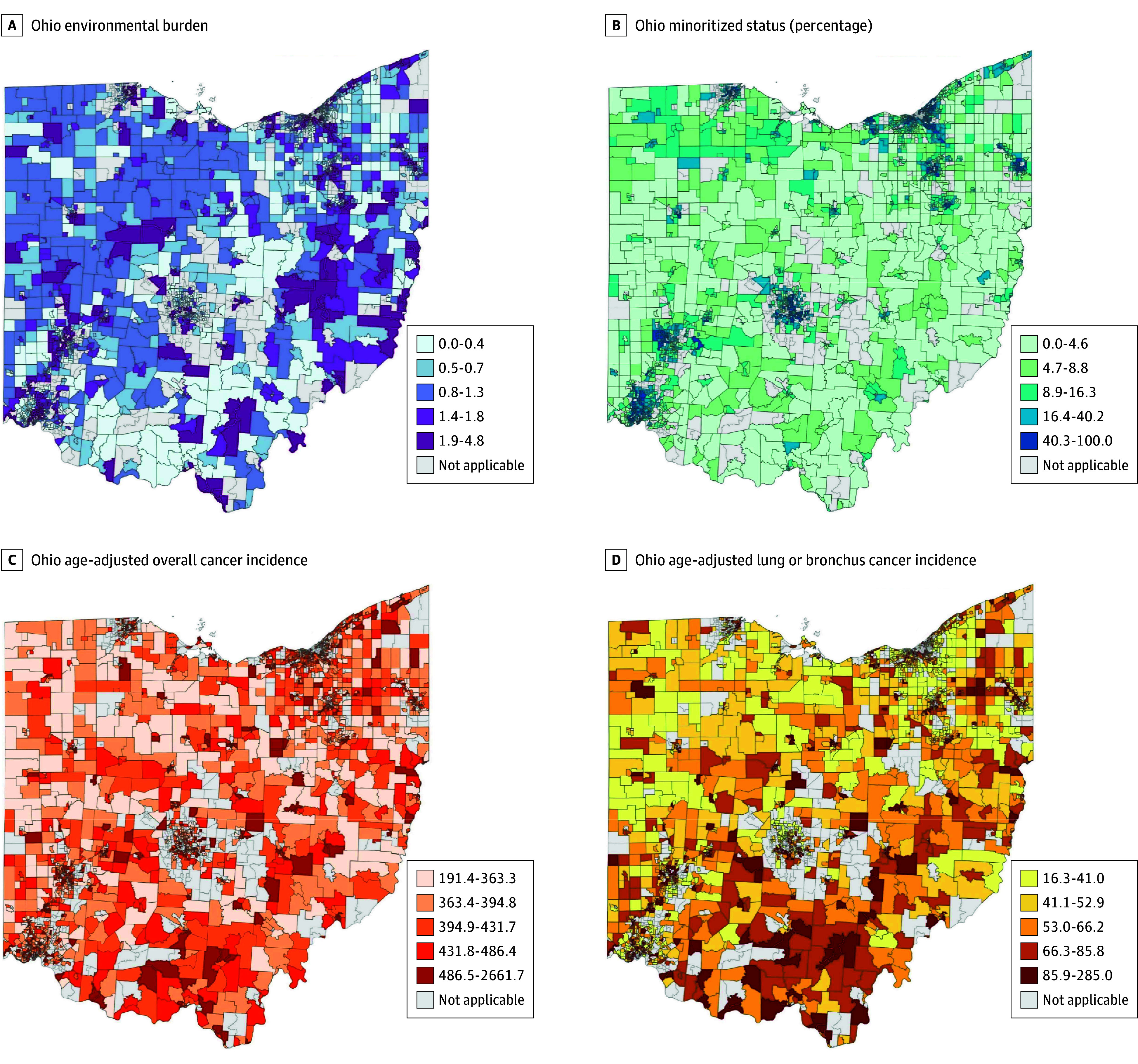
Geographical Distributions of Environmental Burden, Minoritized Status, and Cancer Incidence in Ohio Geographical heatmaps representing the distribution across Ohio, at the census tract level, of A, environmental burden; B, percentage of the population that is racially or ethnically minoritized; C, overall cancer incidence; and D, lung or bronchus cancer incidence. There were 2719 census tracts for all variables except lung or bronchus cancer incidence, for which there were 2510 census tracts. Environmental burden and minoritized status data are from the US Center for Disease Control and Prevention’s Environmental Justice Index database.^15^ Cancer incidence rates are from the Ohio Cancer Incidence Surveillance System database^29^ from 2011 to 2020 and represent the mean annual rate per 100 000, age-adjusted to the 2000 US standard population.

**Figure 3.**
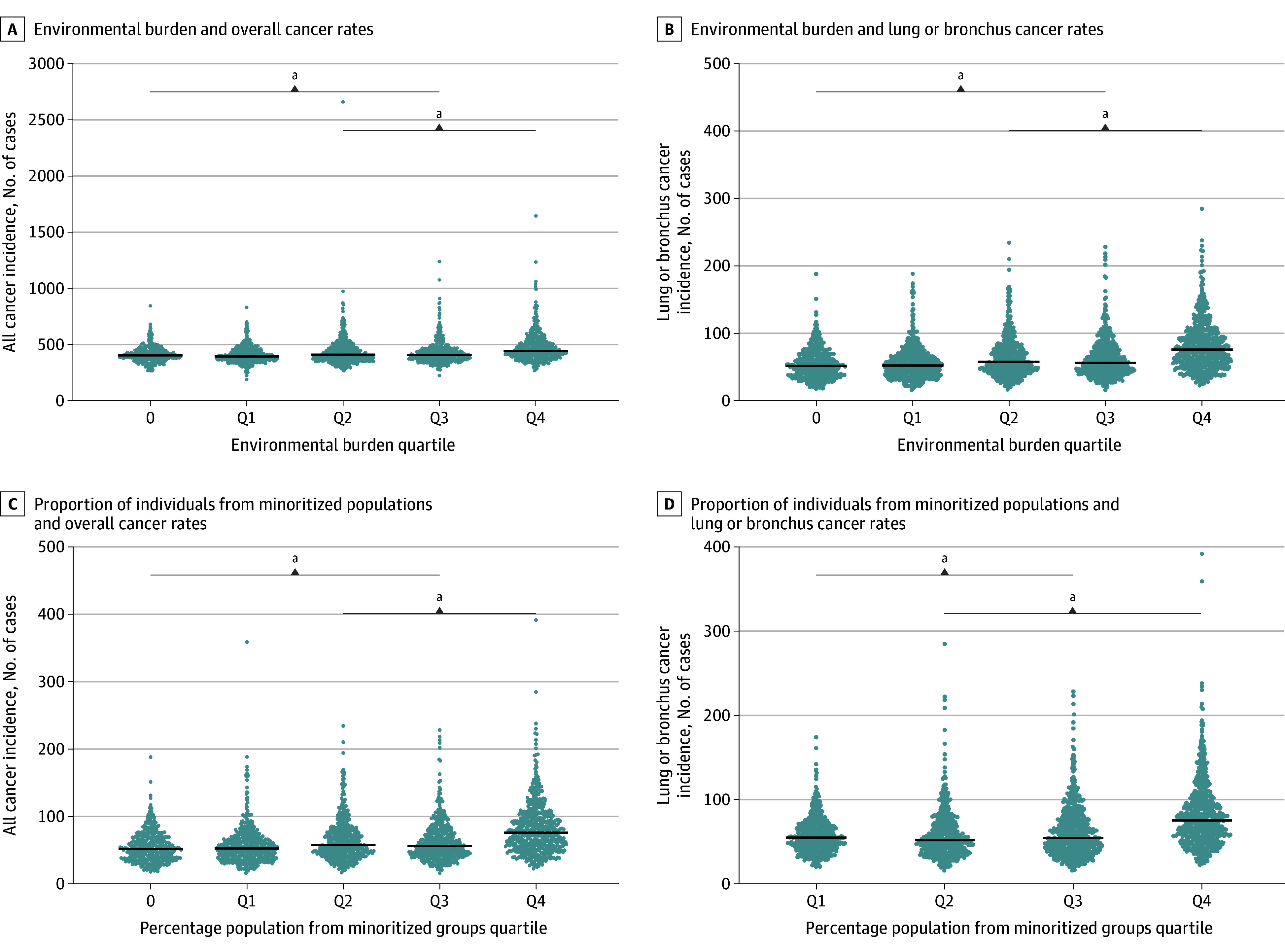
Environmental Burden and Minoritized Status Association With Overall and Lung and Bronchus Cancer Incidence in Ohio A-B, Census tracts were divided into quartiles based on their environmental burden scores (after preserving 0 values), and age-adjusted cancer incidence rates were assessed for A, overall cancer and B, lung or bronchus cancer. Q2-Q4 were significantly greater than baseline (0), and Q4 was significantly higher than all other groups. Each dot represents 1 census tract, and the black lines represent the mean. C-D, Census tracts were divided into quartiles based on the proportion of minoritized individuals in the population, and age-adjusted cancer incidence rates were assessed for C, overall cancer and D, lung or bronchus cancer. Q2-Q4 are significantly greater than Q1, and Q4 is significantly higher than all other groups. Q indicates quartile. ^a^*P* < .05.

We initially linked environmental burden, minoritized status, and cancer incidence to geographic regions in Ohio at the census tract level. Figure 2 shows heatmaps of the geographical distribution of each variable across the state. We could initially observe that higher rates of these variables tended to cluster around major cities and metropolitan areas. To better understand the consequences of each of these variables, we divided them into quartiles and assessed their association with cancer incidence rates. We first examined the association between environmental burden and cancer incidence. Numerous census tracts in Ohio had an environmental burden score of 0, representing a region with low or no known exposures. We treated these areas as the baseline for all possible exposure levels. The remaining tracts with non-0 scores were evenly distributed into quartiles and cancer incidence rates evaluated. For census tracts with an environmental burden score of 0, the mean (SD) age-adjusted overall cancer incidence rate was 414.2 (70.7) cases per 100 000 people. Census tracts with scores in Q2 to Q4 had a statistically significant, greater association between environmental burden and cancer incidence. For those census tracts experiencing the highest levels of environmental exposure in Q4, the mean (SD) cancer incidence rate was observed as 475.9 (129.3) cases per 100 000 people, a rate 14.8% higher than those with a score of 0 (difference, 61.11 cases; 95% CI, 47.89-75.42; *P* < .001) (Figure 3A; eTable 1 in Supplement 1). Similarly, the rate of lung or bronchus cancer was incrementally greater by quartile, from the age-adjusted rate of 55.2 cases per 100 000 people in the tracts with a baseline score of 0. Q4 had a mean (SD) rate of 82.9 (39.5) cases per 100 000 people, representing a 50% higher rate compared with the baseline (difference, 27.7 cases; 95% CI, 23.52-31.88; *P* < .001) (Figure 3B, eTable 1 in Supplement 1).

We likewise divided census tracts into quartiles based on the proportion of the population that is racially or ethnically minoritized, with 0 values included in Q1. Census tracts falling into the Q1 had a mean (SD) age-adjusted overall cancer incidence of 402.5 (66.0) cases per 100 000 people. Census tracts in Q2 to Q4 had a significantly greater overall cancer incidence, with Q4 having a mean (SD) of 483.8 (153.7) cases per 100 000 people, representing 20% greater cancer incidence (difference, 81.27 cases; 95% CI, 70.08-92.45; *P* < .001) (Figure 3C, eTable 2 in Supplement 1). For lung or bronchus cancer, census tracts in Q1 had an age-adjusted mean (SD) incidence rate of 57.8 (21.0) cases per 100 000 people, while in Q4, the rate was 82.6 (40.1) cases per 100 000 people, a 43% higher rate (difference, 24.99 cases; 95% CI, 21.57-28.40; *P* < .001) (Figure 3D, eTable 2 in Supplement 1).

### Joint Association Between Environmental Burden and Minoritized Status on Cancer Incidence Rates in Ohio

**Figure 4.**
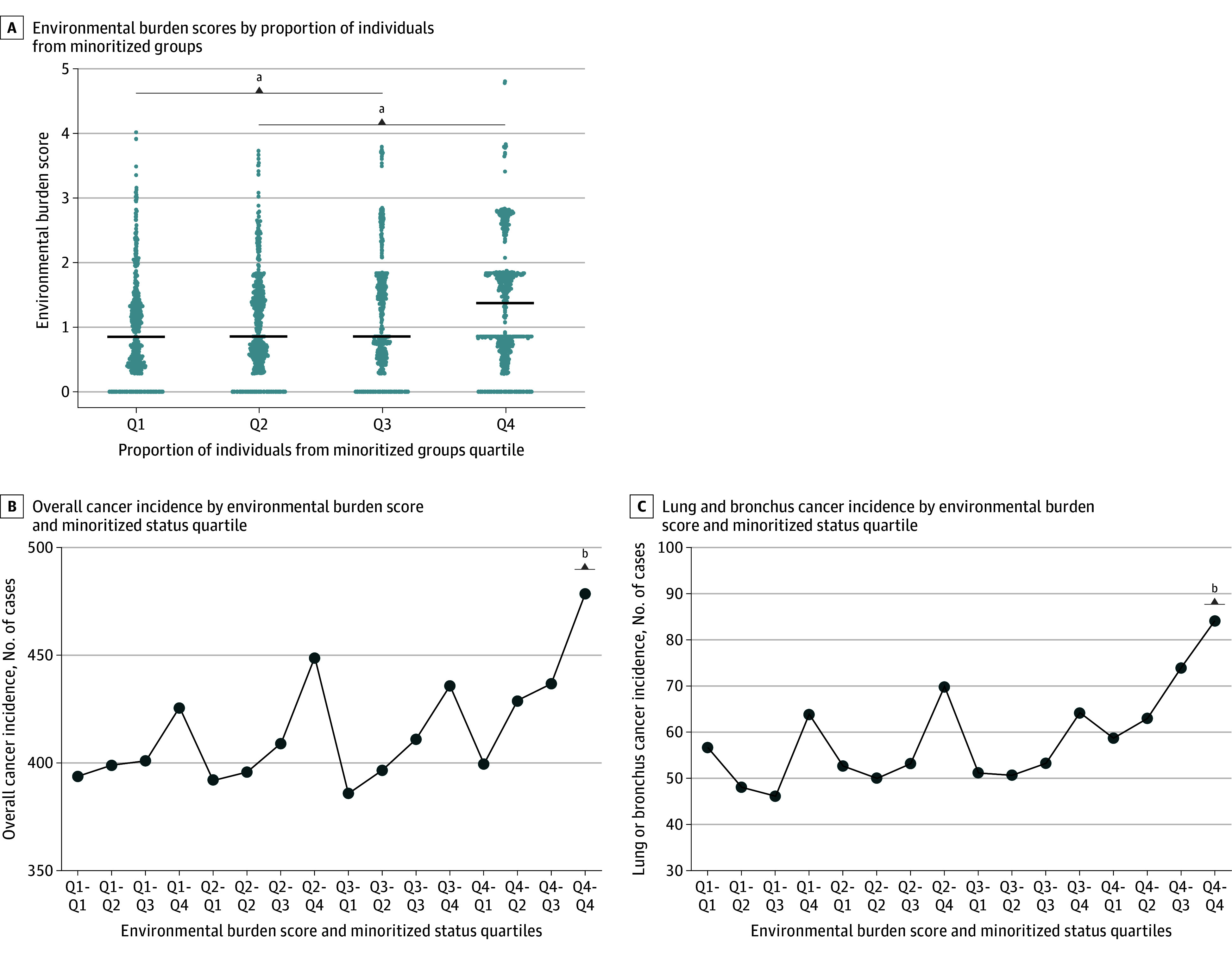
Joint Association Between Environmental Burden and Minoritized Status on Cancer Incidence Rates in Ohio A, Census tracts were divided into quartiles based on the proportion of minoritized individuals in the population and assessed for environmental burden scores. Q2-Q4 are significantly greater than Q1, and Q4 is significantly higher than all other groups. B-C, Environmental burden and minoritized status were divided into quartiles, and census tracts falling into each quartile combination were assessed for incidence rates of B, overall cancer and C, lung or bronchus cancer. Q4-Q4 are statistically greater than all other quartile combinations. Q indicates quartile. ^a^*P* < .05. ^b^*P* < .001.

After understanding the individual associations of environmental burden and minoritized status on cancer incidence, we sought to explore the joint association between these factors. First, we found that those census tracts falling into the highest quartile for minoritized status had a statistically significant higher environmental burden compared with census tracts with a lower proportion of racially and ethnically minoritized individuals (Figure 4A). Next, we divided census tracts into 16 groups based on their quartile rankings for both environmental burden and minoritized status. About 10% of census tracts in the state fell into the top quartiles for both minoritized status and environmental burden (Q4-Q4), and these regions had the highest rates of both overall and lung or bronchus cancer incidence compared with all other groups. Compared with census tracts in the lowest quartiles (Q1:Q1) for both area-level minoritized status and environmental burden, those in the highest quartiles (Q4:Q4) had a 25.9% higher incidence of overall cancer (mean [SD], 508.5 [146.2] per 100 000; 95% CI, 491.3-525.7; vs 425.4 [101.4] per 100 000; 95% CI, 421.4-429.5; *P* < .001) and a 58.9% higher incidence of lung and bronchus cancer (mean [SD], 92.4 [41.6] per 100 000; 95% CI, 87.3-97.5; vs 58.2 [20.9] per 100 000; 95% CI, 55.3-61.0; *P* < .001) (Figure 4B-C).

## Discussion

The analysis reported herein explores the association between environmental burden, minoritized status, and cancer incidence in Ohio neighborhoods. Importantly, investigation of these factors at the census tract level is much more informative than larger geographic regions, such as the county level, because it provides greater clarity into which neighborhoods are particularly affected. Our findings demonstrate that environmental burden and a high proportion of racial and ethnic diversity both were associated with rates of overall and lung or bronchus cancer incidence. Furthermore, communities with a greater proportion of racially and ethnically minoritized individuals had significantly higher environmental burden, suggesting that minoritized individuals may face greater exposures, which could contribute to worse health outcomes. Indeed, those regions with the highest rates of both environmental burden and minoritized status experienced the greatest rates of cancer, both overall and lung or bronchus specifically. This group (Q4-Q4) accounts for approximately 10% of all census tracts in the state, and our results suggest that living in these communities with both high racial and ethnic diversity and high environmental burden results in the worst cancer incidence rates. Expectedly, these census tracts tended to cluster around major cities in Ohio including Cleveland, Columbus, Dayton, Cincinnati, and Toledo, but also in other less populated communities throughout the state.

To investigate environmental burden, we used a subset of data from the CDC’s recently released Environmental Justice Index.^15^ This score is based on proximity to superfund sites, toxic release inventory sites, storage and disposal sites, risk management plan sites, coal mines, and lead mines. This subset was chosen because it is particularly relevant to the state. Ranking twelfth nationwide, Ohio has 37 superfund sites and encompasses portions of Appalachia with high numbers of coal mines. Industry has also brought a number of disposal sites. Our findings suggest that these exposures play a role in cancer incidence rates; however, future studies should more broadly investigate environmental factors to account for variables such as air pollution and built environment as well as discern plausible mechanisms of action for increased cancer incidence.

Environmental and climate-related burden has been an ongoing problem, and studies suggest that tools to mitigate these issues could further perpetuate disparities among populations that are affected by them.^24,25^ To address this, the US government has set a goal that 40% of investments for programs including climate and clean energy will address issues in disadvantaged communities.^26^ Our study identifies regions in Ohio that are most disproportionately burdened and should help ensure resources flow to the communities that are most in need.

### Limitations

In interpreting the study findings, there are several important limitations to consider. While this study leveraged 2 large-scale data sources to examine complex study questions, (EJI and OCISS data), these data sources were examined at the census tract level, not at the individual level. This area-level analysis is prone to ecological fallacy interpretations. However, environmental burden assessments at the level of a person are not easily accessible, and by examining census tracts as opposed to county-level information, we were able to reduce heterogeneity over the unit of observation and to achieve a unit of analysis that more closely represents a neighborhood.

Another potential limitation was the focus on a subset of the EJI data referred to as theme 2 because of its relevance to the state of Ohio. However, the EJI database also captures other variables including ozone, diesel particulate matter, and proximity to railways and roadways. Furthermore, radon, which is not included in the EJI database, is a major risk factor for lung or bronchus cancer, and high risk radon levels occur in approximately 50% of Ohio. While these factors were not evaluated in this analysis, they should be investigated in the future. Another study consideration is the reliance on a dichotomous variable to assess the percentage minoritized population in the census tracts. This approach to assessing racial and ethnic variation was necessary given the variable offered by the EJI metric, which is limited to this categorical variable. However, a valuable use and intent of this variable is to allow for gauging the extent of diversity in a region on study outcomes.

Furthermore, while this study investigates minoritized status, overall, lung or bronchus cancer disproportionately affects Black men in Ohio,^6^ motivating the need for studies that categorically break down race and ethnicity. Additionally, we assess overall cancer incidence and lung or bronchus cancer incidence, but not other cancer types. Generally, a broader analysis of several environmental variables, individual cancer types, and specific racial and ethnic disparities would be informative, and future research should address these questions.

## Conclusions

In this cohort study of census tracts in Ohio, regions exposed to certain environmental contaminants as well as those with high racial and ethnic composition had higher cancer incidence rates. These data suggest an association between environmental burden and area-level minoritized status that may jointly contribute to these elevated rates. In future studies it will be important to examine the potential interplay between these environmental exposures and biological risk factors in driving cancer disparities.^27^ Ultimately, the findings from this study should inform public health policy for cancer prevention and control so that communities at greatest need are prioritized for environmental remediation efforts, which will contribute to greater health equity.

## Notes

Supplement 1. **eTable 1.** Regression Models Were Used to Estimate the Association Between Environmental Burden (EBM) and Rates of Age-Adjusted Overall Cancer or Rates of Age-Adjusted Lung or Bronchus Cancer Incidence **eTable 2. **Regression Models Were Used to Estimate the Association Between Minoritized Status and Rates of Age-Adjusted Overall Cancer or Rates of Age-Adjusted Lung or Bronchus Cancer Incidence

Supplement 2.
Data Sharing Statement
