# Supplementary material for: Cancer Burden in Neighborhoods With Greater Racial Diversity and Environmental Burden
Source: JAMA Netw Open. 2025 Jun 20;8(6):e2516740. doi: 10.1001/jamanetworkopen.2025.16740 (PMC12551422; doi:10.1001/jamanetworkopen.2025.16740)
Supplement: Supplement 1. — eTable 1. Regression Models Were Used to Estimate the Association Between Environmental Burden (EBM) and Rates of Age-Adjusted Overall Cancer or Rates of Age-Adjusted Lung or Bronchus Cancer Incidence eTable 2. Regression Models Were Used to Estimate the Association Between Minoritized Status and Rates of Age-Adjusted Overall Cancer or Rates of Age-Adjusted Lung or Bronchus Cancer Incidence [file jamanetwopen-e2516740-s001.pdf]

Supplemental Online Content

Bobbitt JR, Liu F, Keri RA, Cullen J. Cancer burden in neighborhoods with greater racial diversity and environmental burden. *JAMA Netw Open*. 2025;8(6):e2516740.
doi:10.1001/jamanetworkopen.2025.16740

eTable 1. Regression Models Were Used to Estimate the Association Between Environmental Burden (EBM) and Rates of Age-Adjusted Overall Cancer or Rates of Age-Adjusted Lung/Bronchus Cancer Incidence

eTable 2. Regression Models Were Used to Estimate the Association Between Minoritized Status and Rates of Age-Adjusted Overall Cancer or Rates of Age-Adjusted Lung/Bronchus Cancer Incidence

This supplemental material has been provided by the authors to give readers additional information about their work.

© 2025 Bobbitt JR et al. *JAMA Network Open*.

eTable 1. Regression Models Were Used to Estimate the Association Between Environmental Burden (EBM) and Rates of Age-Adjusted Overall Cancer or Rates of Age-Adjusted Lung/Bronchus Cancer Incidence

	Adjusted rate of overall cancer		Adjusted rate of lung/bronchus cancer	
	**Estimate (95% CI)**	**p-value**	**Estimate (95% CI)**	**p-value**
**EBM – Q1***	-4.39 [-18.15, 9.38]	0.532	2.03 [-2.15, 6.21]	0.342
**EBM – Q2***	20.47 [6.70, 34.25]	0.004	9.68 [5.50, 13.86]	<0.001
**EBM – Q3***	14.51 [0.74, 28.28]	0.039	7.91 [3.73, 12.09]	<0.001
**EBM – Q4***	61.66 [47.89, 75.42]	<0.001	27.7 [23.52, 31.88]	<0.001

*Referent category is EBM-0

eTable 2. Regression Models Were Used to Estimate the Association Between Minoritized Status and Rates of Age-Adjusted Overall Cancer or Rates of Age-Adjusted Lung/Bronchus Cancer Incidence

	Adjusted rate of overall cancer		Adjusted rate of lung/bronchus cancer	
	**Estimate (95% CI)**	**p-value**	**Estimate (95% CI)**	**p-value**
**Minority – Q2***	14.7 [3.51, 25.90]	0.010	1.02 [-2.40, 4.44]	0.557
**Minority – Q3***	30.10 [18.89, 41.30]	<0.001	5.09 [1.66, 8.51]	0.003
**Minority – Q4***	81.27 [70.08, 92.45]	<0.001	24.99 [21.57, 28.40]	<0.001

*Referent category is Minority-Q1

© 2025 Bobbitt JR et al. *JAMA Network Open*.
