# Supplementary material for: Cancer Burden in Neighborhoods With Greater Racial Diversity and Environmental Burden
Source: JAMA Netw Open. 2025 Jun 20;8(6):e2516740. doi: 10.1001/jamanetworkopen.2025.16740 (PMC12551422; doi:10.1001/jamanetworkopen.2025.16740)
Supplement: Supplement 2. — Data Sharing Statement [file jamanetwopen-e2516740-s002.pdf]

Data Sharing Statement

Bobbitt. Cancer Burden in Neighborhoods With Greater Racial Diversity and Environmental Burden. *JAMA Netw Open*. Published June 20, 2025.
doi:10.1001/jamanetworkopen.2025.16740

Data
Data available: No

Additional Information
Explanation for why data not available: Researchers must get approval from the Ohio Department of Health to access cancer incidence data from the Ohio Cancer Incidence Surveillance System. The environmental data from the CDC is already publicly available.
